# Tea polyphenols attenuate liver inflammation by modulating obesity-related genes and down-regulating COX-2 and iNOS expression in high fat-fed dogs

**DOI:** 10.1186/s12917-020-02448-7

**Published:** 2020-07-08

**Authors:** Sajid Ur Rahman, Yingying Huang, Lei Zhu, Xiaoyan Chu, Shahid Ahmed Junejo, Yafei Zhang, Ibrar Muhammad Khan, Yu Li, Shibin Feng, Jinjie Wu, Xichun Wang

**Affiliations:** 1 College of Animal Science and Technology, Anhui Agricultural University, 130 West Changjiang Road, Hefei, 230036 China; 2 School of Tea and Food Technology, Anhui Agricultural University, 130 West Changjiang Road, Hefei, 230036 China; 3 Anhui Provincial Laboratory of Local Livestock and Poultry Genetical Resource Conservation and Breeding, Anhui Agricultural University, 130 West Changjiang Road, Hefei, 230036 China

**Keywords:** Tea polyphenols, Obesity, COX-2 expression, Dog, Inflammatory cytokines

## Abstract

**Background:**

Tea polyphenols (TPs) attenuate obesity related liver inflammation; however, the anti-obesity effects and anti-inflammatory mechanisms are not clearly understood. This study aimed to determine whether the anti-obesity and anti-inflammatory TPs mechanisms associated with cyclooxygenase-2 (COX-2) and inducible nitric oxide synthase (iNOS) expression levels, and obesity-related gene response in dogs.

**Results:**

Dogs fed TPs displayed significantly decreased (*p <* 0.01) mRNA expression of tumor necrosis factor-α (TNF-α), interleukin-1 beta (IL-1β), and interleukin-6 (IL-6) compared to dogs that consumed high-fat diet (HFD) alone. TPs significantly (*p <* 0.01) inhibited COX-2 and iNOS expression level, and decreased liver fat content and degeneration.

**Conclusion:**

These results suggested that TPs act as a therapeutic agent for obesity, liver inflammation, and fat degeneration via COX-2 and iNOS inhibition, with TNF-α, IL-1β, and IL-6 involvement.

## Background

Obesity is related to systemically present low-grade inflammation, and white adipose tissues that are defined as the abnormal accumulation of excessive fats in adipose tissue resulting from an imbalance between energy intake and expenditure [1, 2]. Moreover, obesity is characterized by changes of circulating hormones including Ghrelin, thyroid stimulating hormone and free triiodothyronine levels [3, 4], and nutrients including glucose and fatty acids, as well as other metabolic changes [5]. Obesity occurrence has increased globally over the past decades. Recently, obesity has a significant impact on humans, as it contributes to the development of metabolic and cardiovascular diseases [6]. The most effective treatment for obesity prevention is dietary control and physical exercise-based therapies combined with pharmacological therapies, as well as bariatric surgery, but different lifestyles do not allow most people access to proper obesity therapy [7, 8]. In contrast, many pharmacological therapies are available for obesity treatment, but their outcome is very limited and can involve many side effects [9]. Therefore, controlling obesity, a major cause of inflammation and cancer, with natural products and herbs has become increasingly popular [10].

Cyclooxygenase-2 (COX-2) is a vital enzyme involved in arachidonic acid metabolization [11]. In normal cell biology, the expression of COX-2 is low or unobservable, but during response to different stimuli, such as cytokines and growth factors, it quickly increases [12]. Nuclear factor-ĸB (NF-ĸB) has been shown to play an important role in the regulation of cellular COX-2 expression [13]. COX-2 expression is regulated at both the transcriptional and posttranscriptional level, and COX-2 and inducible nitric oxide synthase (iNOS) are the two main inflammatory mediators for inflammation and cancer [14]. Numerous upstream signaling pathways, including mitogen-activated protein kinases and phosphatidylinositol 3 kinase pathways, have been involved COX-2 expression regulation at various levels [15].

**Fig. 1:**
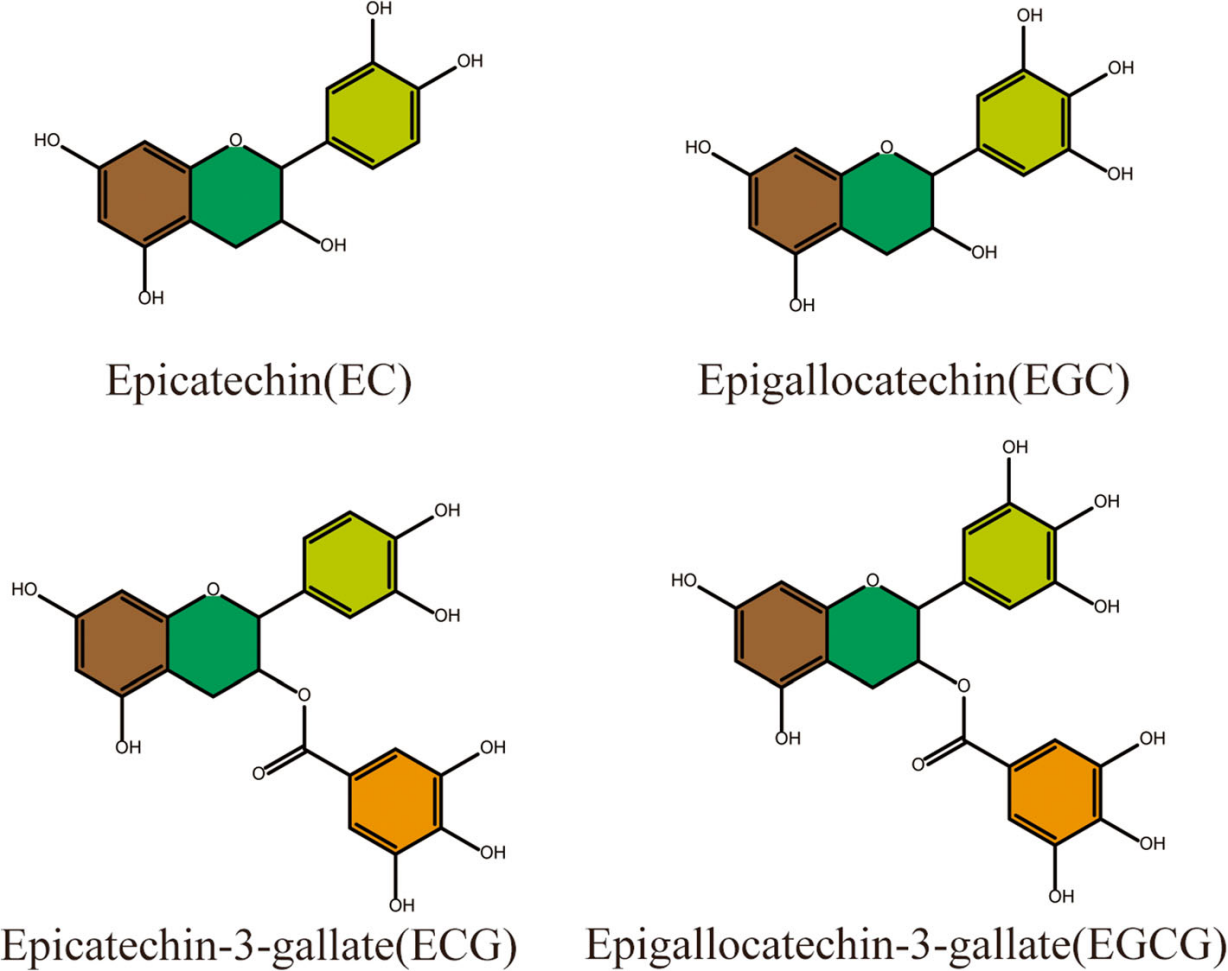
Polyphenolic components of TPs

Green tea and its major polyphenolic components (EGCG (−) epigallocatechin gallate; EGC (−) epigallocatechin; ECG (−)-epicatechin gallate; EC (−)-epicatechin) catechins (Fig. 1) possess many potential health benefits including anti-inflammatory, antioxidant, anti-carcinogenic, and cardio-protective effects [16–19]. Many studies highlight the beneficial effects of TPs on increasing energy expenditure and decreasing fat mass, as well as helping in weight maintenance after weight control programs [20]. The catechin content of green tea is high, and may prevent a number of chronic disorders when ingested regularly, including protective effect in autoimmune disorders such as Sjogren’s syndrome, uveitis, rheumatoid arthritis, and experimental autoimmune encephalomyelitis [21]. TPs consumption has also increased fat oxidation, suppressed adipocyte differentiation and proliferation, and inhibited fat absorption [22, 23]. Research on the relationship between tea ingestion and obesity has suggested that tea polyphenols exert anti-obesity properties by stimulating hepatic lipid metabolism and thermogenesis, which prevent gastric and pancreatic lipases as well as modulating appetite [24]. However, tea consumption-induced variations in serum and hepatic metabolites associated with obesity and obesity-related dysfunction are not obviously implicit, despite an accumulation of physiological data.

Hepatic steatosis, also called non-alcoholic fatty liver disease (NAFLD), is a common pathological change in the liver which, together with low-grade inflammation, is related to obesity and metabolic disorders [25, 26]. TPs protect against NAFLD by decreasing lipid accumulation in the liver and injury in HFD C57BL/6 J obese mice. A long period of EGCG treatment reduced obesity development, and symptoms related to metabolic diseases and fatty liver through reduced lipid absorption and decreased inflammatory cytokines such as TNF-α, IL-1β, and IL-6 [27].

In this study, we investigated whether the anti-obesity and anti-inflammatory properties of TPs are associated with the inhibition of COX-2 and iNOS expression through the modulation of signaling pathways that regulate COX-2 gene expression with pro-inflammatory cytokines response as well as symptoms of metabolic syndrome and inflammation in the liver of dogs after fed an HFD or an HFD combined with TPs.

## Results

### Effects of TPs and HFD on BW

**Fig. 2:**
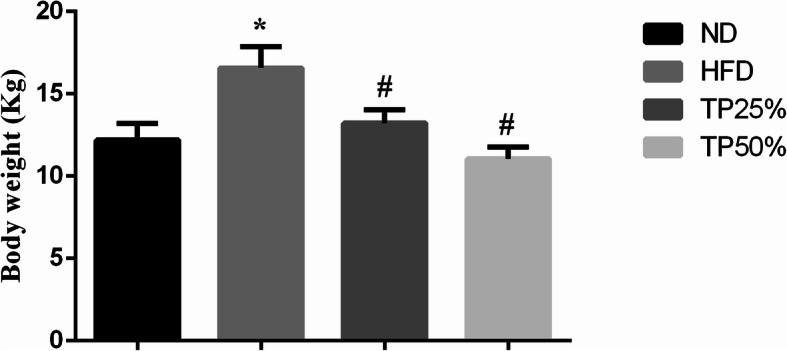
Effects of TPs on body weight during the 12 week treatment period. Body weight was higher in the HFD group compared to the TPs treatment groups. Values are expressed as the mean ± SD. * *p <* 0.05 vs. ND group; # *p <* 0.05 vs. HFD group

The body weight (BW) of HFD fed dogs resulted in a 3.37 kg increase (12.20 ± 1.0 to 16.57 ± 1.3 kg), differing significantly (*p <* 0.05) from the ND group (Fig. 2). In contrast, the TPs25% and TPs50% groups exhibited lower weight gain than the HFD group. Comparison of the TPs50% group with the HFD group showed a significant (*p <* 0.05) BW reduction from 16.57 ± 1.3 to 11.60 ± 0.7 kg after TPs treatment.

### Effect of TPs on Lee index, liver weight, and liver coefficient

**Fig. 3:**
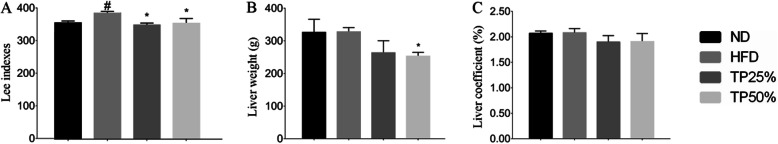
Effects of TPs on Lee index, liver weight, and liver coefficient during the 12 week treatment period. Note: Compared to the ND group, data from the HFD group marked with # indicated a significant difference at *p <* 0.05; compared to the HFD group, data from either TPs group marked with * indicated significance at *p <* 0.05

The Lee index of the HFD group (8.33%) was significantly higher than that of the ND group (*p <* 0.05). Compared to the HFD group, the Lee index of the TPs25% and TPs50% groups show a significant (*p <* 0.05) reduction of 9.42 and 8.17%, respectively presented in Fig. 3a. These results indicated that the addition of 25 and 50% TPs to an HFD inhibited an increased Lee index. Figure 3b showed that the liver weight of the HFD group was 0.31% higher than that of the ND group, but the difference was not significant (*p >* 0.05). The TPs25% and TPs50% groups displayed a liver weight reduction of 19.48 and 22.67%, respectively, when compared to the HFD group. The value of the TPs50% group was significantly lower than that of the HFD group (*p <* 0.05). These results indicated that the addition of 25 and 50% TPs in an HFD inhibited diet-induced increasing canine liver weight. As shown in Fig. 3c, the liver coefficient of the HFD group was 0.64% higher than that of the ND group, but the difference was not statistically significant (*p >* 0.05). The TPs25% and TPs50% groups showed an 8.81 and 8.29% decrease, respectively, when compared with the HFD group. These results demonstrated that adding 25 and 50% tea polyphenols to an HFD inhibited an increased liver coefficient, but did not alter the liver coefficient.

### Effect of TPs on serum biochemical parameters

The data in supplementary Tables S1-S4 showed that, at week 0 of the experimental trials, there was no significant difference between total cholesterol (TC), total glyceride (TG), low-density lipoprotein cholesterol (LDL-C) and high-density lipoprotein cholesterol (HDL-C) level of all the groups (*p >* 0.05). After 12 weeks of treatment, the serum TC of the HFD group was significantly (*p <* 0.05) higher than that of the ND group, 41.70 and 17.34%, respectively. Compared to the HFD group, the TC level of the TPs25% and TPs50% groups decreased by 6.7 and 11.19%, respectively, but the difference was not significant (*p >* 0.05) presented in supplementary Table S1. During week 0 of the experimental trials, the serum TG level in all groups was similar; however, at week 12th, the serum TG of the HFD group was significantly higher than that of the ND group (*p <* 0.05), 93.34 and 28.89%, respectively. Compared with the HFD group, the addition of 25 and 50% TPs significantly decreased (*p <* 0.05) the TG level in serum by 13.79 and 17.24%, respectively (Supplementary Table S2). There was no significant difference (*p >* 0.05) in serum LDL-C between the HFD group and the ND group or both TPs groups at week 0 (Supplementary Table S3). At week 12th, the serum LDL-C of the HFD group was significantly (*p <* 0.05) higher than that of the ND group, 52.63 and 6.25%, respectively. The 25 and 50% TP treatment groups showed 20.83 and 26.08% lower LDL-C levels than that of the HFD group, respectively. Supplementary Table S4 showed that the serum HDL-C levels of the dogs in each group were similar during week 0; and there was no significant difference between the HFD and ND groups (*p >* 0.05). At week 12th, the serum HDL-C was significantly lowered in the HFD group than in the ND group (*p <* 0.05). The HDL-C level was reduced to 14.34% in the HFD group, and increased 27.57% in the ND group. The percentage was reduced to 26.77% when compared both the HFD and ND groups. The HDL-C level was higher 16.30 and 22.46%, respectively in both TP groups than in the HFD group, but the difference was non-significant (*p >* 0.05).

### TPs inhibit COX-2 and iNOS expression in liver tissue

**Fig. 4:**
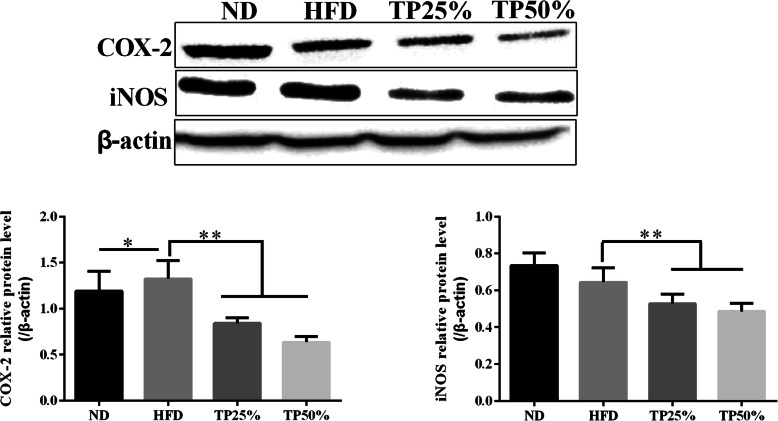
Effects of TPs and an HFD on COX-2 and iNOS expression level. Identical loading was confirmed by re-probing the membrane with β-actin. All values were indicated as the mean ± SEM. *, (*p <* 0.05) and **, (*p <* 0.01). The experiment was repeated three times with similar results

Oral supplementation of TPs inhibited COX-2 expression in dogs liver tissue when compared with HFD group, which displayed significantly increased COX-2 expression (*p <* 0.05). Further investigation to determine whether iNOS associated with the TPs mediated suppression of nitric oxide (NO) production showed that iNOS levels were significantly inhibited (*p <* 0.05) in the TPs50% group after 12 weeks of treatment (Fig. 4).

### Effects of TPs on inflammatory cytokines mRNA expression

**Fig. 5:**
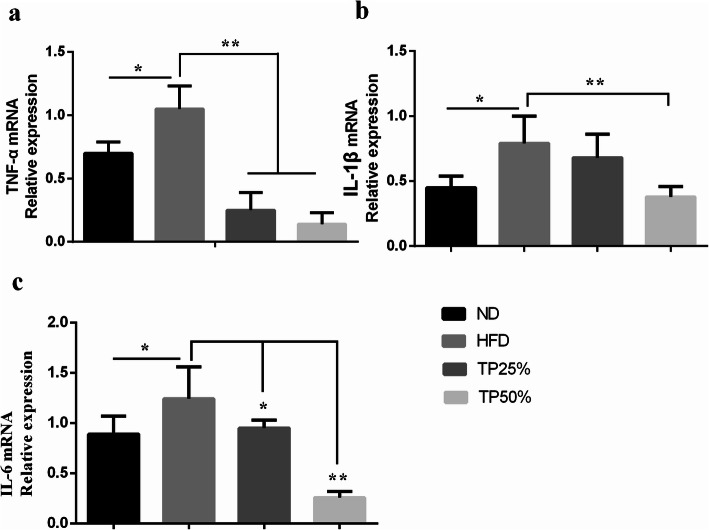
Effect of an HFD and TPs supplementation on relative *TNF-α*, *IL-1β*, and *IL-6* mRNA expression levels in liver tissue. Relative mRNA expression levels were determined by qRT-PCR. Data were presented as means ± SD. Values with *, (*p <* 0.05) and **, (*p <* 0.01) were considered statistically significant difference between groups

Levels of TNF-α, IL-1β, and IL-6 inflammatory cytokines produced by liver tissue are presented in Fig. 5a-c. Inflammatory cytokine mRNA expression significantly increased (*p <* 0.05) in the HFD group compared to the ND group. However, the TPs25% and TPs50% groups showed a significantly decreased expression (*p <* 0.01) compared to the HFD group throughout the entire experimental period, and a greater reduction was seen in the TPs50% group.

### Histological liver and adipose tissue analysis

**Fig. 6:**
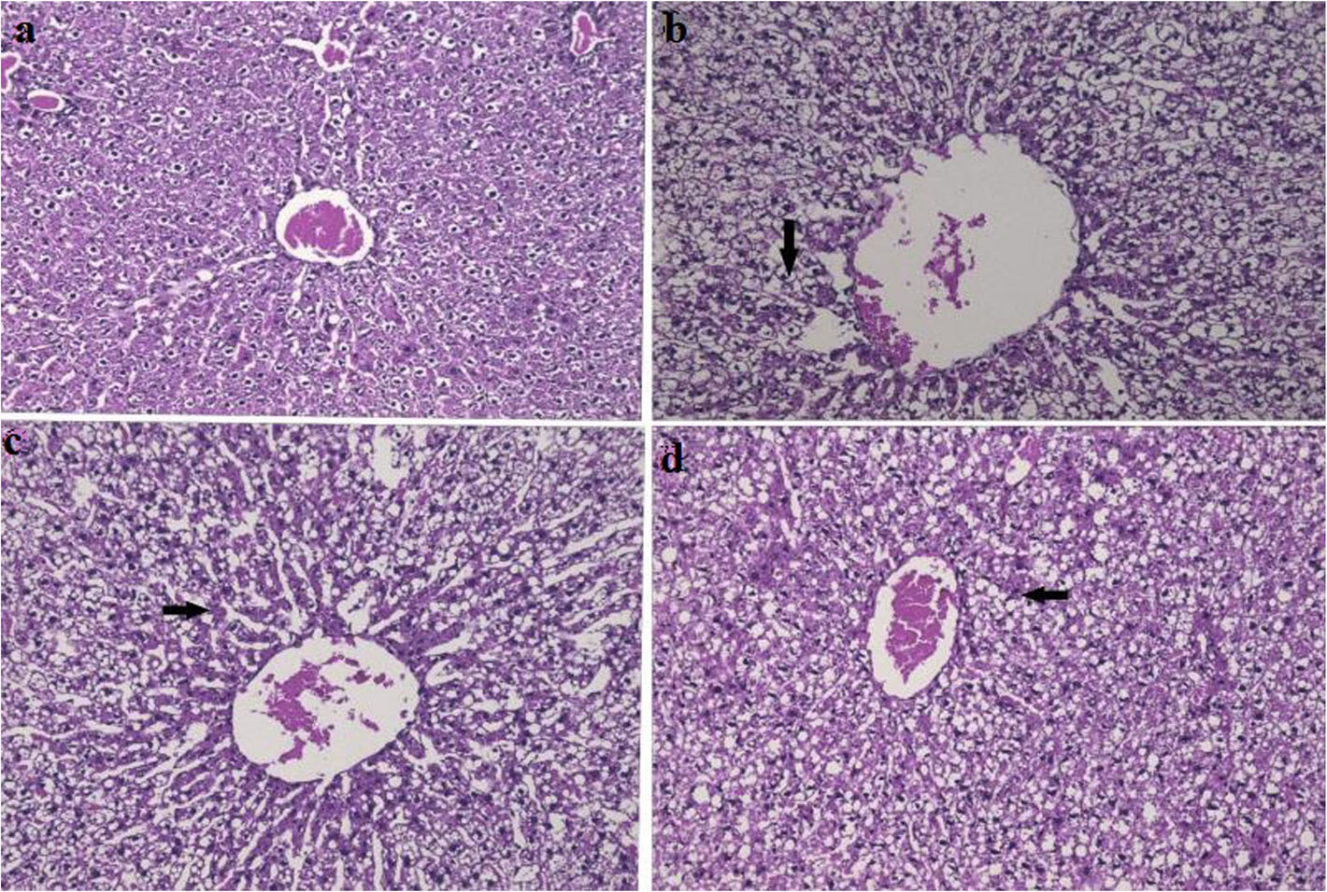
Histological examination of dog’s liver [Magnification 100X; H&E staining]: **a** normal diet (ND) group; **b** high-fat diet (HFD) group; **c** tea polyphenols 25% (TPs25%) group; **d** tea polyphenols 50% (TPs50%) group. The black arrows indicate fat droplets

In the hematoxylin and eosin (H&E) staining examination, pathological liver symptoms were examined in the liver of HFD dogs. The hepatic cells were observed to be seriously collapsed; the number of fat droplets and distorted cells was significantly larger. However, the ND group exhibited fine architectural characteristics, a low volume of fatty cells and no fat degeneration. The dogs fed with TPs25%, the degeneration of the liver cells was less and the area of fatty cells was decreased than in the HFD group. The TPs50% was found to be the most effective, as shown in Fig. 6a-d).

**Fig. 7:**
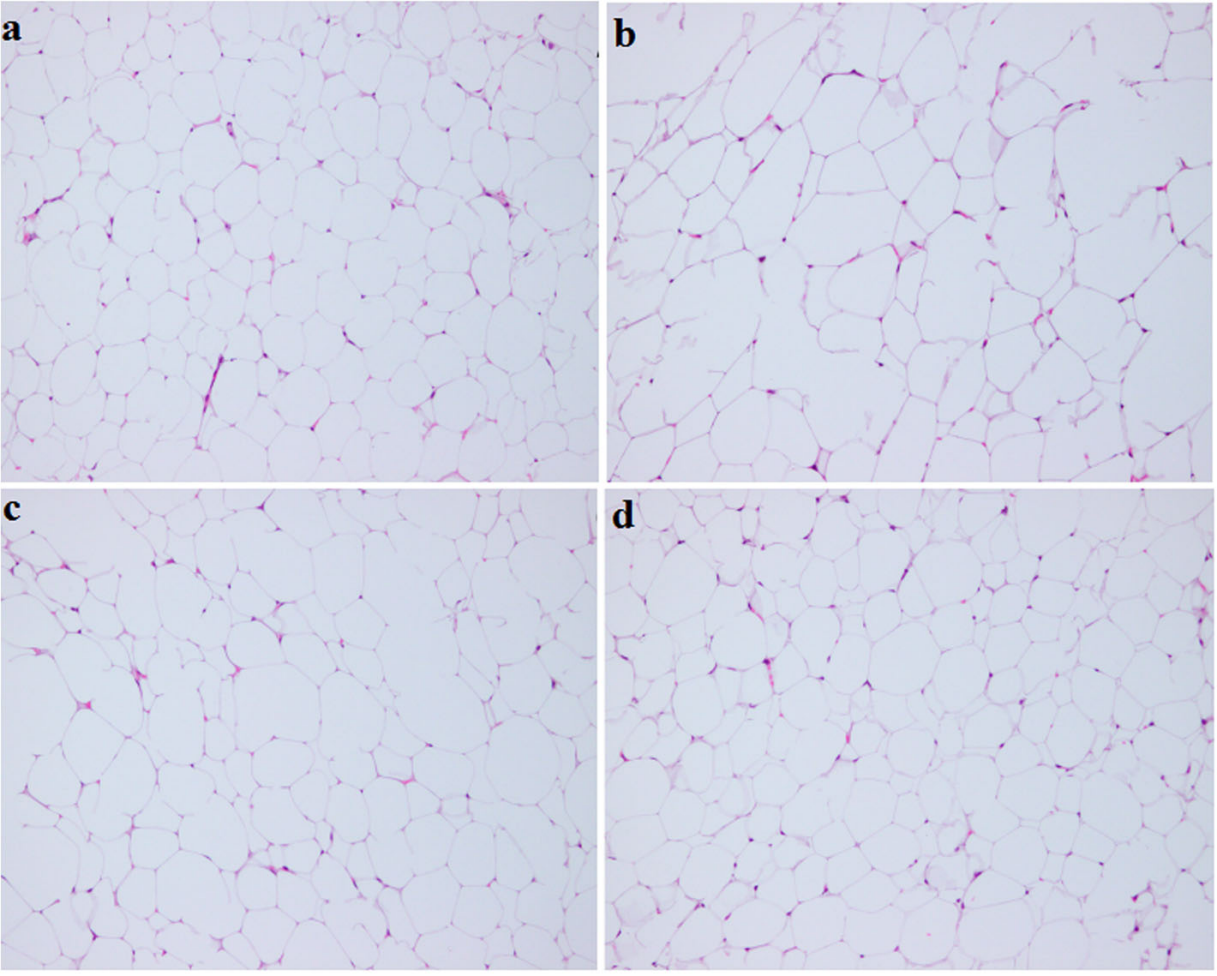
Histological examination of dog’s adipose tissue [Magnification 100X; H&E staining]: **a** normal diet (ND) group; **b** high fat diet (HFD) group; **c** tea polyphenols 25% (TPs25%) group; **d** tea polyphenols 50% (TPs50%) group

As shown in (Fig. 7a, b), the size and the volume of white fat cells were considerably larger in the HFD group than in the ND group. By contrast, the size and volume of fat cells were reduced in TPs25% and TPs50%, compared to the HFD group (Fig. 7c, d).

## Discussion

Numerous experimental and epidemiological studies have associated tea consumption with several biological and pharmacological functions [28, 29]. The present study demonstrated that an HFD intake induced dog’s obesity, indicators of fatty liver disease, and inflammatory cytokine expression. In contrast, both TPs administrations showed a BW reduction, decreased indicators of fatty liver disease, and reduced inflammatory cytokine expression with expression level inhibition of some genes. Many studies have revealed that an HFD significantly increases adipose tissue accumulation, owing to increasing energy availability that leads to increased BW [30, 31].

In the current research, both TPs treatments exerted beneficial effects against obesity. BW gain was significantly decreased in TPs groups (25 and 50%), resulting in a reduction of liver fatty acid as well as inhibiting the increased in canine liver weight and Lee index seen in the HFD group. Similar previously reported results [32] demonstrate that an HFD induces more severe diseases than an ND and that EGCG treatment can alleviate these symptoms, as well as reduce BW and body fat accumulation. The effects of green tea and TPs have been examined in a number of animal obesity models. Powdered green tea (130 mg) administered to male Zucker rats fed a diet containing 50% sucrose and 15% butter very quickly result in a BW reduction [33]. In our study, we administered TPs25% and TPs50% for 12 weeks, and found a significant BW reduction from 16.57 ± 1.3 HFD alone to 13.50 ± 2.0 TPs25% and 11.60 ± 0.7 kg TPs50%, while the dogs fed an HFD increased their BW from 13.20 ± 1.0 ND to 16.57 ± 1.3 kg (*p <* 0.01; Fig. 2). These results are further supported by another study [34], in which green tea treatment (2% in the diet) reduced body fat accumulation in Sprague-Dawley rats after 14 days, but did not alter BW gain. In the present study, the BW was measured by digital balance and not the body condition score or body fat index is because the large dogs were difficult to handle as well as the dogs used in the present study were non-cooperative during the experimental trials.

There have been numerous epidemiological and experimental examinations of the effects of TPs on plasma and serum lipid profiles [35–37], with inconsistent outcomes. For example, in a rodent study, Sayama and co-workers [38] found that green tea lowered triacylglycerol (TAG) levels but did not change TC concentrations. The previous study has shown that the severity of EGCG induced toxicity seems to be a function of dose, administration route and period of treatment [39, 40]. Dietary dosages of green tea extract can improve lipid profile and insulin sensitivity and change the genes expression involved in the homeostasis of glucose and lipid [41]. However, Ashida and colleagues [42] stated that green tea supplementation reduced TC, HDL-cholesterol, LDL-cholesterol, and non-esterified fatty acid levels without changing TAG concentrations. The present study found a marked decrease in serum TC and TG concentrations in the TP groups related to decreasing LDL-C levels in dogs. However, no significant difference was observed in HDL-C level.

**Fig. 8:**
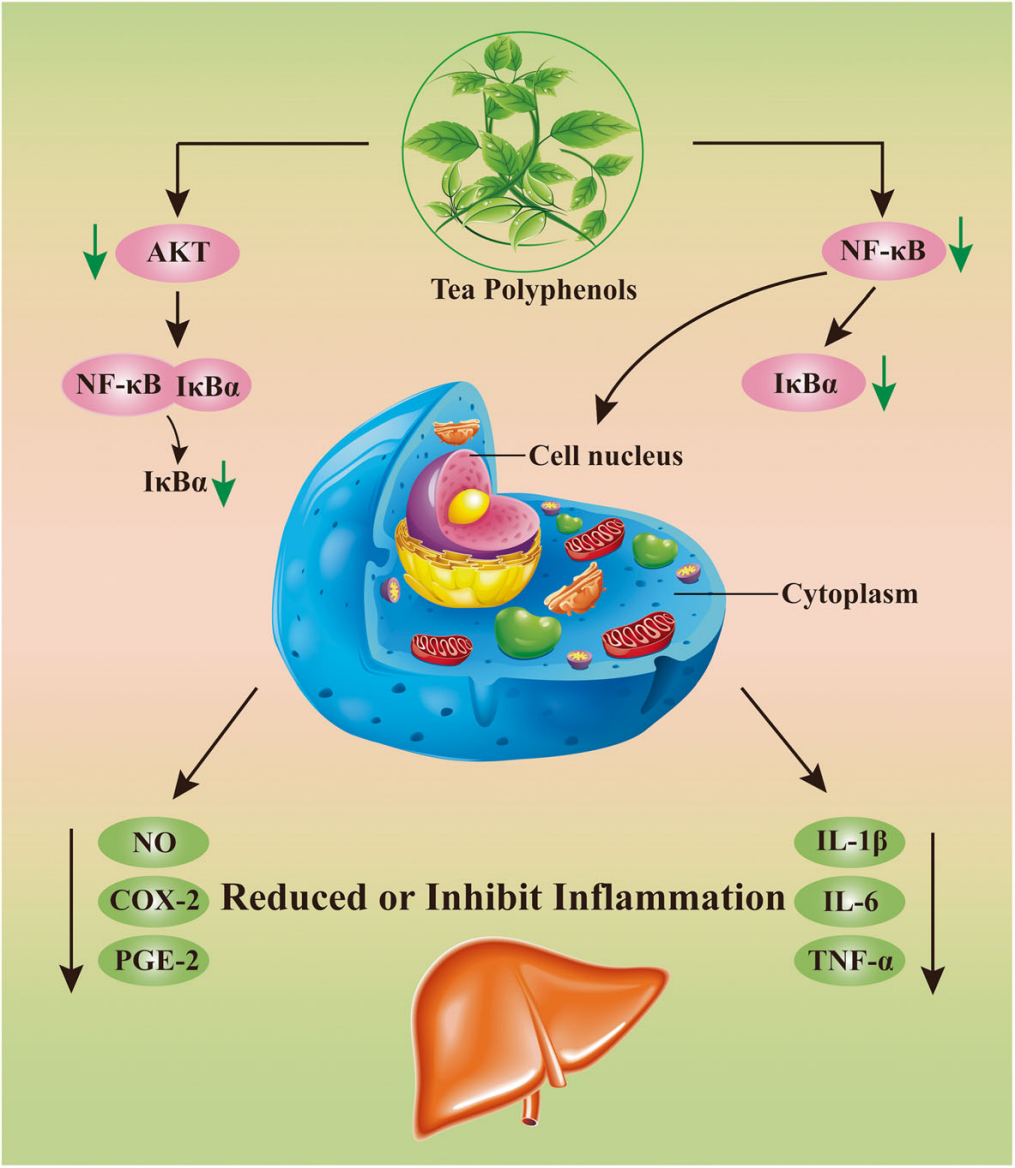
Proposed mechanism of protection by TPs in an HFD induced obese dog’s liver model. ↓ Designate downregulation or decreased expression of protein. AKT, Ak transforming; IKBα, I-kappa-B-alpha; NF-kB, nuclear factor- kappa B; NO, nitric oxide; COX-2, cyclooxygenase-2; PGE-2, prostaglandin E2

COX-2 levels are an important tool for detecting inflammatory diseases and inhibiting cancer [43]. Increased COX-2 expression is found in a wide array of inflammatory conditions and human cancers, including lung and liver cancers [44]. One study shows that increased COX-2 expression is related to tumor invasion [45]. Our results indicate that TPs decreased COX-2 and iNOS expression in canine liver tissues. Extreme and continued NO generation produced by increased iNOS expression has been involved in inflammation and tumorigenesis, while COX-2 facilitated prostaglandin production has been revealed to trigger cell proliferation, angiogenesis, and invasion in cancer improvement [46]. Meanwhile, chronic inflammation is inclined to malignancy; the inhibition of COX-2 by TPs seems likely to contribute to both the anti-obesity and anti-inflammatory responses induced by an HFD. The proposed mechanism for this protection is discussed in Fig. 8.

Numerous animal studies indicate that excessive body fat negatively affects the immune function of obese individuals [47, 48]. Furthermore, alterations in the immune function of obese animals are considered to be significant in producing pathophysiological obesity effects. Green tea supplementation in a study showed that, of all of the major green tea components, catechins were responsible for the suppression of BW gain and adipose tissue accumulation; however, catechin supplementation (0.3% of diet) in mice did not change these parameters [49, 50]. In this study, the anti-obesity and anti-inflammatory effects of TPs were evaluated by various approaches in a dog model. Elevated pro-inflammatory cytokines have been shown to play a significant pathogenic role in liver inflammation. In this study, we found that TNF-α expression was highly increased (*p <* 0.05) in the HFD group compared to the ND group after 12 weeks, but expression was reduced in the TPs25% and TPs50% groups. IL-6 and IL-1β mRNA expression also significantly decreased (*p <* 0.01) in both TPs groups compared to the HFD group. In our previous study, we also demonstrated that *TNF-α*, *IL-6* and *IL-1β* mRNA gene expression were significantly decreased by green tea polyphenols in the intestinal epithelial layer of canines [18]. These results suggested the protective effects of TPs on immunological liver inflammation act by inhibiting inflammatory cytokine expression.

A non-alcoholic fatty liver disease is the hepatic expression of a metabolic syndrome [51]. During this disease, the patient has a higher risk of vascular disorders and coronary disease because of the primary metabolic illness [52]. An increased mortality rate was found in the patients with cirrhosis symptoms. The accumulation of fats in the liver is usually related to a cluster of metabolic diseases [53]. An HFD has previously caused metabolic diseases by increasing fat oxidation and decreasing fat storage because of high satiety [54]. We demonstrated that dogs fed an HFD showed collapsed hepatic cells and increased number of fat droplets, compared to ND group which displayed normal architectural characteristics. Furthermore, TPs decreased the area of fatty cells with less degeneration of the liver cells. Interestingly, these results support the previous study conducted by Han et al. in a mice model feed with dietary fibers [55]. In the present study, the TPs treatments enhanced liver fat reduction and reduced liver inflammation by reducing pro-inflammatory cytokine expression. The previously reported consumption of an HFD was shown to contribute to obesity and increase the risk of NAFLD in obese individuals [56].

## Conclusions

These findings indicate the potential of TPs in suppressing liver inflammation and fat degeneration induced by an HFD. We demonstrated that TPs down-regulate COX-2 and iNOS expression levels with an inflammatory cytokine response in liver tissues. This indicates that TPs supplementation could provide a therapeutic agent for the treatment of obesity and obesity-related inflammation by reducing weight gain and liver weight, and inhibiting fat degeneration via anti-oxidant action. Our research findings provide novel insights that may be useful to the fields of obesity and liver inflammation management, both of which would benefit from an effective treatment with no known side effects.

## Methods

### Samples preparation

The polyphenolic components, EGC, EGCG, GCG, and ECG (Table 1), present in tea were detected via a photodiode array of high-performance liquid chromatography for rapid TP determination, purity > 98.50%) provided by the Wuhu Tianyuan Science and Technology Development Co., Ltd., Anhui Province, China. TPs were separated on a C18 RP-column by gradient elution using mixed solutions of mobile phase A and B in different quantities. Mobile phase A was a solution of methanol and formic acid mixed in the ratio of 99.7 to 0.3 by volume. Mobile phase B was a formic acid solution, containing 3 vol of formic acid in 1 l of solution. The flow rate of mobile phase was set at 1.0 ml/min, the column temperature was 40 °C, and the UV-detection wavelength was 280 nm.

**Table 1:** The major contents of TPs used in this experiment (purity> 98.50%)

Compound	GA	GC	EGC	C	THF	EC	EGCG	GCG	ECG	CAF
Content (mg/g)	0.16	0.32	13.21	0.54	0.74	4.51	42.40	1.12	6.18	1.26

*GA* gallic acid, *GC* (−)-gallocatechin, *EGC* (−)-epigallocatechin, *C* (+)-catechin, *THF* tetrahydrofuran, *EC* (−)-epicatechin, *EGCG* (−)-epigallocatechin gallate, *GCG* (−) gallocatechin gallate, *ECG* (−)-epicatechin gallate, *CAF* caffeine

### Animals and feed intake

The experimental protocol was approved by the Anhui Agricultural University Animal Care and Institutional Animal Ethical Committee (ZXD-C2018815), Hefei, China. Sixteen clinically healthy male beagle dogs (13–14 months old) with mean BW of 12.09 ± 0.4 kg, and identical parity were purchased from Jiangsu Yadong Experimental Animal Research Institute Co., Ltd. The dogs were individually housed in different rooms inside cages in the animal hospital of Anhui Agricultural University, Hefei, China under controlled conditions (15–20 °C, 98% relative humidity, and a 12 h light-dark cycle) for the entire experimental period. Male dogs were selected to reduce distinctions in diet consumption due to ovarian hormones, in addition to their growing faster than females [57]. The dog’s food tray was used to feed the dogs twice a day, in the morning and evening by mixing of TPs and HFD in their standard chow. The BW was measured weekly via digital balance. Diet composition and major components are described in Table 2.

**Table 2:** Major components g/kg and the composition of diet used in this study

Ingredient g/kg	
Water%	≤100
Crude protein%	≥200
Crude fat%	≥45
Crude fibe%	≤40
Crude ash%	≤90
Calcium%	7–10
Total phosphorus%	5–8
Calcium: total phosphorus%	1.2:1–1.4:1
Lysine g/100 g	≥7.4
Methionine-cystine g/100 g	≥5.4
Normal Dog food (dog Chow)	76%
Egg yolk	10%
Pig Oil	10%
Cholesterol	2.5%
Bile acid Sodium	1.5%
Total	100%

### Obesity induction and TPs treatment

The dogs were initially maintained on a normal diet (ND) for a 4 week acclimatization period (3.885 kcal/day, 39.4% carbohydrates, 32.2% fats, and 28.4% protein). After acclimatization, the dogs were randomly divided into four groups, ND, HFD, TPs25%, and TPs50% (four dogs per group). The first two groups were fed ND standard chow or an HFD, respectively, both served as control groups until the end of study. The remaining two groups were co-administered either low dose (TPs25%; 0.25 g/kg BW) or high dose (TPs50%; 0.50 g/kg BW) TPs with an HFD. The ND group was continually fed commercial normal chow, while the HFD supplementation consisted of 5530 kcal/day (27.4% carbohydrates, 53.0% fats, and 19.6% proteins) for 12 weeks, as this period is sufficient to observe significant increases in adiposity and BW [58]. The HFD was formulated and administered as previously reported [59], with some modification. Dogs with 10% BW above the maximum BW of ND dogs were considered obese after HFD administration [60].

### Sample collection

At the end of the trials (12 weeks), all dogs were anesthetized with xylazine hydrochloride (1.5 mg/kg, IM), and (20 mg/kg, IV) thiopental sodium (Abbott). Blood samples were collected from the cephalic vein, and serum was separated and kept at − 80 °C for further analysis. After blood sampling collection, all dogs were euthanized with a lethal dose of thiopental injection. Liver sample was collected with microscopic slides at 0 °C, frozen on dry-ice, and stored at − 80 °C until analysis of inflammatory cytokines and protein expression by quantitative real-time PCR or western blotting.

### Calculation of Lee index, liver weight, and their coefficient

The Lee index and liver coefficient were calculated by measuring dog length from the nasal cavity to the anal cavity, and the body weight. The following formulae were applied to calculate the Lee index and liver coefficient:

1 $$ \mathrm{Lee}\ \mathrm{index}\ \left(\mathrm{Obesity}\ \mathrm{assessment}\ \mathrm{index}\right)=\sqrt[3]{~} \left(\mathrm{weight}\ \left(\mathrm{g}\right)/\right(\mathrm{body}\ \mathrm{length}\ \left(\mathrm{cm}\right)\times 103 $$

2 $$ \mathrm{Liver}\ \mathrm{coefficient}=\mathrm{hepatic}\ \mathrm{weight}\ \left(\mathrm{g}\right)\div \mathrm{dog}\ \mathrm{weight}\ \left(\mathrm{g}\right)\times 100\% $$

### Determination of serum biochemical indicators

Blood samples were subsequently collected biweekly after all dogs had fasted overnight. Samples were taken under ketamine and xylazine anesthesia and during the end of the induction, and treatment period. The collected blood was centrifuged for 10 min at 3500 rpm at room temperature, and the serum was collected and stored at − 80 °C for biochemical analysis. TC was determined by the cholesterol oxidase method and TG was determined by the GPO-PAP method. The LDL-C and HDL-C were determined using the scavenging method. The assay kits for these methods were purchased from Elabscience Biotechnology Co., Ltd.

### Western blot analysis

The collected liver samples were washed twice with cold phosphate-buffered saline at 800×g for 10 min. Total protein was extracted using a radio-immunoprecipitation assay buffer and the protein concentration was measured by the BCA method. The proteins were separated by 10% SDS-PAGE and transferred to polyvinylidene difluoride membranes (Shanghai Jinsheng Biological Engineering Co., Ltd). The membranes were blocked with 50% bovine serum albumin containing 0.05% Tween-20 in Tris buffered-saline for 4 h at room temperature. After 4 h, the polyvinylidene difluoride membrane was incubated with the primary antibody for the whole night. The membranes were then washed three times (5 min each), and incubated with secondary antibodies for 45 min at room temperature. The protein bands for COX-2, iNOS, and β-actin (Elabscience Biotechnology Co., Ltd) were identified with a chemiluminescence western blot detection system (Bio-Rad, Hercules, CA, USA).

### Quantitative real-time PCR (qRT-PCR) assay

The expressions of inflammatory cytokines (TNF-α, IL-1β, and IL-6) in the liver were measured by qRT-PCR. Total RNA was extracted from liver tissue using Trizol reagent (Takara, Dalian, China). The reverse transcription system (TaKaRa, Dalian, China) was used to reverse transcribe the total RNA. The relative mRNA expression levels of inflammatory cytokines were measured with SYBR Green (TaKaRa, Dalian, China) in an ABI 7900 Fast Real-Time PCR system thermal cycler (Applied Biosystems). Each sample was amplified by primer and reference gene primer respectively, and each sample was assayed three times. The amplification system (20 μl) was established according to the manufacturer’s instructions. The following program was used for qRT-PCR: pre-denaturation (95 °C, 15 min), 40 cycles of denaturation (95 °C, 10 s), annealing (55 °C, 20 s), extension (72 °C, 32 s), and fluorescence signal acquisition. The primer sequences for IL-1β, IL-6, and TNF-α are shown in Table 3. The mRNA expression of each gene was normalized to glyceraldehyde 3-phosphate dehydrogenase (*GAPDH*). Statistical analyses were performed based on ΔCt (Ct_target gene_ – Ct_GAPDH_). Data are presented as 2^−ΔΔCt^*.*

**Table 3:** Sequences of the oligonucleotide primer used for quantitative real-time PCR

Genes	Primer F	Primer R
*IL-1β*	TGAAGTGCTGCTGCCAAG	GGAAGAGAATTCCATGGT
*IL-6*	ACCAGGAACGAAAGAGAG	GGAATGCCCATGAACTAC
*TNF-α*	CTTCTCCTTCCTCCTCGT	AGCCCTTAATTCTCTTTC
*GAPDH*	GGAGAAAGCTGCCAAATATG	ACCAGGAAATGAGCTTGACA

### Liver and adipose tissue histopathology

The liver and adipose tissue (subcutaneous abdominal region) samples collected from all groups were fixed in 0.01 M phosphate-buffered 10% formalin solution. The tissues were fixed, and then embedded in paraffin. Histological sections 5 μm thick were cut and stained with H&E for histopathological examination.

### Statistical analysis

The data were analyzed with SPSS 17.0 statistical software (SPSS Inc., Chicago, IL, USA), and Graph Pad Prism statistics software package, version 6.0, for Windows (Grappa Software, San Diego, CA, USA). The data were presented as the mean ± SD. Statistical differences were established by one-way analysis of variance. Quantity one software (Bio-Rad) was used to analyze the protein band intensity. Values were considered statistically significant when *p <* 0.05, or highly significant when *p <* 0.01.

## Supplementary information

**Additional file 1. Table S1. **Effects of HFD and TPs on serum TC of dogs (mmol/L). **Table S2.** Effects of HFD and TPs on serum TG of dogs (mmol/L). **Table S3.** Effects of HFD and TPs on serum LDL-C of dogs (mmol/L). **Table S4.** Effects of HFD and TPs on serum HDL-C of dogs (mmol/L).

## Data Availability

The data used and/or analyzed during the current study are available from the corresponding author on reasonable request.

## Abbreviations

TPs: Tea polyphenols
COX-2: Cyclooxygenase-2
iNOS: Inducible nitric oxide synthase
TNF-α: Tumor necrosis factor-α
IL-1β: Interleukin-1 beta
NF-ĸB: Nuclear factor-ĸB
NAFLD: Non-alcoholic fatty liver disease
BW: Bodyweight
TC: Total cholesterol
TG: Total glyceride
LDL-C: Low-density lipoprotein cholesterol
HDL-C: High-density lipoprotein cholesterol
qRT-PCR: Quantitative real-time polymerase chain reaction
GAPDH: Glyceraldehyde 3-phosphate dehydrogenase
