# Supplementary material for: Tea polyphenols attenuate liver inflammation by modulating obesity-related genes and down-regulating COX-2 and iNOS expression in high fat-fed dogs
Source: BMC Vet Res. 2020 Jul 8;16:234. doi: 10.1186/s12917-020-02448-7 (PMC7346471; doi:10.1186/s12917-020-02448-7)
Supplement: Supplementary file 1 — Additional file 1. Table S1. Effects of HFD and TPs on serum TC of dogs (mmol/L). Table S2. Effects of HFD and TPs on serum TG of dogs (mmol/L). Table S3. Effects of HFD and TPs on serum LDL-C of dogs (mmol/L). Table S4. Effects of HFD and TPs on serum HDL-C of dogs (mmol/L). [file 12917_2020_2448_MOESM1_ESM.docx]

**Supplementary data**

**Tea polyphenols attenuate liver inflammation by modulating obesity-related genes and down-regulating COX-2 and iNOS expression in high fat-fed dogs**

Sajid Ur Rahman^1^, Yingying Huang^1^, Lei Zhu^1^, Xiaoyan Chu^1^, Shahid Ahmed Junejo^2^, Yafei Zhang^1^, Ibrar Muhammad Khan^3^, Yu Li^1^, Shibin Feng^1^, Jinjie Wu^1^ and Xichun Wang^*1^

^1^College of Animal Science and Technology, Anhui Agricultural University, 130 West Changjiang Road, Hefei 230036, China

^2^School of Tea and Food Technology, Anhui Agricultural University, 130 West Changjiang Road, Hefei 230036, China

^3^Anhui Provincial Laboratory of Local Livestock and Poultry Genetical Resource Conservation and Breeding, Anhui Agricultural University, 130 West Changjiang Road, Hefei 230036, China

*** Correspondence:**

Xichun Wang

E-mail address: wangxichun@ahau.edu.cn

Tel: +86- 13865984965

Fax: +86- 551 65785027

**Supplementary information**

The supplementary information includes 4 supplementary tables

**Supplementary Tables**

**Table S1** Effects of HFD and TPs on serum TC of dogs (mmol/L)

| Weeks | ND | HFD | TP25% | TP50% |
| --- | --- | --- | --- | --- |
| 0 | 3.98±0.28 | 4.10±0.83 | 3.87±0.48 | 4.03±1.14 |
| 2 | 3.96±0.26 | 4.69±0.66 | 4.28±0.22 | 4.34±0.34 |
| 4 | 4.16±0.10 | 4.67±0.62 | 4.47±0.46 | 4.44±0.75 |
| 6 | 4.36±0.25 | 5.20±0.19^*^ | 4.87±0.50 | 4.96±1.07 |
| 8 | 4.50±0.27 | 5.64±0.20^*^ | 4.99±0.29 | 5.13±1.01 |
| 10 | 4.44±0.32 | 5.23±0.63 | 5.07±0.86 | 5.09±0.86 |
| 12 | 4.67±0.22 | 5.81±0.33^*^ | 5.42±0.66 | 5.16±0.66 |

Note: Compared with ND group data of high-fat diet group with star ^*^ indicates significance difference at (*p<*0.05) level. The same rules are applying also for tables S2, S3 and S4.

**Table S2** Effects of HFD and TPs on serum TG of dogs (mmol/L)

| Weeks | ND | HFD | TP25% | TP50% |
| --- | --- | --- | --- | --- |
| 0 | 0.45±0.13 | 0.45±0.04 | 0.45±0.10 | 0.43±0.10 |
| 2 | 0.48±0.08 | 0.53±0.12 | 0.55±0.09 | 0.53±0.08 |
| 4 | 0.51±0.07 | 0.60±0.13 | 0.55±0.09 | 0.49±0.04 |
| 6 | 0.49±0.06 | 0.54±0.02 | 0.57±0.01 | 0.51±0.12 |
| 8 | 0.52±0.01 | 0.65±0.10 | 0.59±0.09 | 0.60±0.14 |
| 10 | 0.59±0.14 | 0.76±0.07 | 0.66±0.04^#^ | 0.61±0.01^##^ |
| 12 | 0.58±0.05 | 0.87±0.12^*^ | 0.75±0.05 | 0.72±0.03 |

* *p<*0.05, ** *p<*0.01 vs. ND group; # *p<*0.05, ## *p<*0.01 vs. HFD group

**Table S3** Effects of HFD and TPs on serum LDL-C of dogs (mmol/L)

| Weeks | ND | HFD | TP25% | TP50% |
| --- | --- | --- | --- | --- |
| 0 | 0.16±0.02 | 0.19±0.06 | 0.17±0.05 | 0.17±0.07 |
| 2 | 0.15±0.05 | 0.26±0.05^*^ | 0.20±0.03 | 0.22±0.10 |
| 4 | 0.19±0.06 | 0.22±0.06 | 0.21±0.06 | 0.18±0.06 |
| 6 | 0.17±0.07 | 0.25±0.08 | 0.22±0.06 | 0.26±0.26 |
| 8 | 0.16±0.05 | 0.28±0.05^*^ | 0.21±0.05^#^ | 0.20±0.02^##^ |
| 10 | 0.15±0.01 | 0.25±0.03^*^ | 0.24±0.06 | 0.21±0.05 |
| 12 | 0.17±0.05 | 0.29±0.01^*^ | 0.24±0.02 | 0.23±0.05 |

Compared with ND group data of HFD group with star * indicates significance difference at (*p<*0.05) level; Compared with HFD group data of different concentrations of TPs added groups with hash # shows significance difference at (*p<*0.05), and double hash ## designates significant difference at (*p<*0.01) level.

**Table S4** Effects of HFD and TPs on serum HDL-C of dogs (mmol/L)

| Weeks | ND | HFD | TP25% | TP50% |
| --- | --- | --- | --- | --- |
| 0 | 2.43±0.17 | 2.65±0.34 | 2.42±0.36 | 2.42±0.54 |
| 2 | 2.47±0.11 | 2.87±0.39 | 2.68±0.10 | 2.61±0.15 |
| 4 | 2.65±0.18 | 2.92±0.19^*^ | 2.93±0.28 | 2.97±0.69 |
| 6 | 2.86±0.37 | 2.76±0.32 | 2.80±0.25 | 2.60±0.03 |
| 8 | 3.09±0.45 | 2.70±0.52 | 2.79±0.17 | 2.86±0.11 |
| 10 | 3.12±0.74 | 2.46±0.32 | 2.66±0.36 | 2.66±0.37 |
| 12 | 3.10±0.21 | 2.27±0.39^*^ | 2.64±0.43 | 2.78±0.17 |

* *p<*0.05, ** *p<*0.01 shows comparison of HFD with ND group.
